# Naphthalenones and Depsidones from a Sponge-Derived Strain of the Fungus *Corynespora cassiicola*

**DOI:** 10.3390/molecules21020160

**Published:** 2016-01-28

**Authors:** Dong-Lin Zhao, Chang-Lun Shao, Chao-Yi Wang, Mei Wang, Lu-Jia Yang, Chang-Yun Wang

**Affiliations:** 1 Key Laboratory of Marine Drugs, the Ministry of Education of China, School of Medicine and Pharmacy, Ocean University of China, Qingdao 266003, China; lanseyimi@sina.com (D.-L.Z.); shaochanglun@163.com (C.-L.S.); chaoyi0411@126.com (C.-Y.W.); caoyuxiaowu@163.com (M.W.); yangljshw@163.com (L.-J.Y.); 2 Institute of Evolution & Marine Biodiversity, Ocean University of China, Qingdao 266003, China

**Keywords:** sponge-derived fungus, *Corynespora cassiicola*, naphthalenone, depsidone, cytotoxicity

## Abstract

Two new naphthalenones, corynenones A and B (**1** and **2**), and one new depsidone, corynesidone E (**3**), together with one known depsidone, corynesidone A (**4**) and two known diphenyl ethers, corynethers A (**5**) and B (**6**), were isolated from the sponge-derived fungus *Corynespora cassiicola* XS-20090I7. Their structures including absolute configurations were determined by spectroscopic data and electronic circular dichroism (ECD) spectra. Compounds **4** and **5** showed cytotoxicity against human promyelocytic leukemia HL-60 and human cervical carcinoma HeLa cell lines.

## 1. Introduction

**Figure 1:**
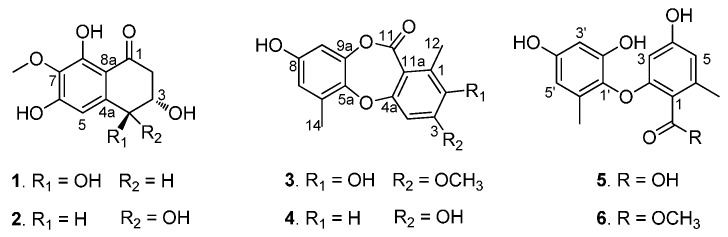
Compounds **1**–**6** isolated from the sponge-derived fungus *Corynespora cassiicola*.

Marine fungi have played an important role for drug discovery as a source of structurally unique and biologically active secondary metabolites. Among marine fungi, sponge-derived fungi take up a large proportion of marine fungal diversity, and produce numerous new bioactive compounds which display promising biological and pharmacological properties, such as antiviral, antibacterial, antitumor, antifouling, anti-inflammatory, and immunomodulatory activities [1,2,3,4]. Recently, we reported 12 new chromone derivatives isolated from the sponge-derived fungus *Corynespora cassiicola* XS-200900I7, collected from the Xisha Islands coral reef in the South China Sea [5], which encouraged us to continue to study this fungal strain. The further chemical investigation of this strain led to the isolation of two new naphthalenones, corynenones A and B (**1** and **2**), and one new depsidone, corynesidone E (**3**), together with three known compounds, corynesidone A (**4**) [6], corynethers A (**5**) [6] and B (**6**) [7] (Figure 1). Herein we report the isolation, structure elucidation, and biological activities of these compounds.

## 2. Results

**Figure 2:**
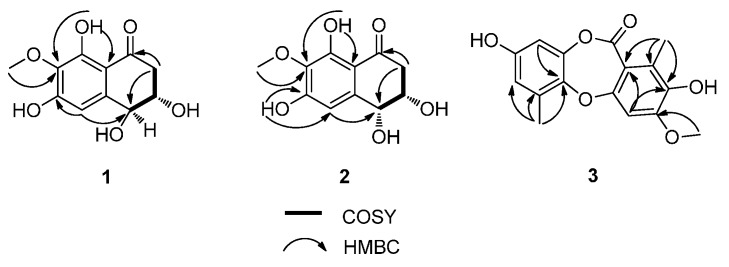
COSY Correlations and Key HMBCs of **1**, **2**, and **3**.

**Figure 3:**
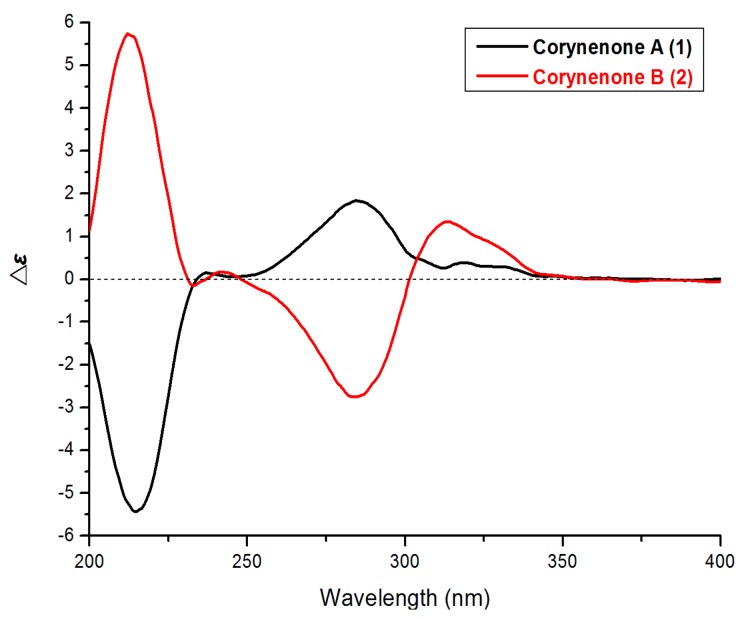
ECD spectra of **1** and **2**.

Corynenone A (**1**) was isolated as a brown amorphous powder with the molecular formula of C_1__1_H_12_O_6_, for which six degrees of unsaturation were required. The ^1^H-NMR spectrum exhibited signals for one hydrogen-bonded phenolic hydroxy group at δ_H_ 12.93 (s), one aromatic proton at δ_H_ 6.73 (s), two oxygenated methine protons at δ_H_ 4.54 (d, *J* = 7.5 Hz) and δ_H_ 4.01 (m), and one methoxy group at δ_H_ 3.81 (s), together with one set of nonequivalent methylene protons at δ_H_ 2.94 (dd, *J* = 17.0, 4.0 Hz) and δ_H_ 2.65 (dd, *J* = 17.0, 9.0 Hz). In the ^13^C-NMR and DEPT spectra, five of the 11 carbons required by the molecular formula were identified as protonated centers, including three methines (one aromatic carbon, two oxygenated carbons) at δ_C_ 107.4, 73.1 and 71.4, one methoxy group at δ_C_ 60.5, and one methylene at δ_C_ 44.4. The six nonprotonated centers were separated into two categories: an α,β-unsaturated ketone at δ_C_ 202.1, five aromatic carbons at δ_C_ 157.9, 157.3, 142.7, 134.2, and 110.8. These spectroscopic features revealed that the parent skeleton of **1** was naphthalenone, and was very similar to 4-hydroxyscytalone previously isolated from *Pyricularia oryzae* Cavara [8]. The significant difference between these two compounds was the presence of a methoxy group signal in the ^1^H- and ^13^C-NMR spectra of **1**. The downfield shift of C-7 in the ^13^C-NMR indicated that the methoxy group was attached to C-7, which was confirmed by the HMBC correlations from 7-OCH_3_ to C-7 and from H-5 to C-4 (Figure 2). Detailed assignments for proton and carbon signals (Table 1) were accomplished by analysis of 1D- and 2D-NMR data. The relative configurations of C-3 and C-4 were straightforwardly elucidated as *trans*-arrangement based on the ax/ax coupling constant of the methine proton H-4 (δ_H_ 4.54) coupled to H-3 (δ_H_ 4.01) with a value of 7.50 Hz [8,9], which suggested a half-chair conformation of the cyclohexenone ring. The absolute configuration of **1** was determined by comparison its ECD spectrum with those of 3,4-diol naphthalenones described in the literature. For such naphthalenones, the first positive Cotton effect around 285 nm and second negative effect around 215 nm correspond to a 4*S* configuration, whereas the first negative and second positive ones reflect a 4*R* configuration [9,10]. Therefore, 4*S* configuration was designed for **1** by its first positive and second negative Cotton effects at 280 and 214 nm (Figure 3). Furthermore, based on the relative configuration, the absolute configuration of **1** was determined as 3*S*,4*S*.

**Table 1:** ^1^H- and ^13^C-NMR Data (500 and 125 MHz, resp. δ in ppm, *J* in Hz) of compounds **1** and **2**.

Position	1 (Acetone-*d*_6_)		2 (DMSO-*d*_6_)	
	δ(H)	δ(C)	δ(H)	δ(C)
1		202.1		201.8
2	2.94 (dd, *J* = 17.0, 4.0) 2.65 (dd, *J* = 17.0, 9.0)	44.4	2.81 (dd, *J* = 17.0, 3.0) 2.66 (dd, *J* = 17.0, 5.5)	43.4
3	4.01, m	71.4	4.10, brs	69.1
4	4.54( d, *J* = 7.5)	73.1	4.61( d, *J* = 3.0)	69.2
4a		142.7		141.9
5	6.73, s	107.4	6.59, s	107.2
6		157.9		157.4
7		134.2		133.0
8		157.3		156.4
8a		110.8		109.2
3-OH			4.97, s	
4-OH			5.39, brs	
6-OH			10.45, s	
7-OCH_3_	3.81, s	60.5	3.69, s	59.7
8-OH	12.93, s		12.81, s	

Corynenone B (**2**) was also obtained as a brown amorphous powder and has a molecular formula C_1__1_H_12_O_6_ as deduced from its HRESIMS spectrum. Detailed analysis of the ^1^H- and ^13^C-NMR as well as 2D-NMR spectra of **1** and **2** (Table 1, Figure 2) revealed that these two compounds showed high similarity in NMR spectroscopic data. The significant difference between **1** and **2** was the chemical shifts of the oxymethines at C-3 and C-4 (δ_C_ 69.1 and 69.2 in **1** *vs.* δ_C_ 71.4 and 73.1 in **2**) in the ^13^C-NMR spectra. This NMR feature implied that **1** and **2** might be diastereoisomers differing from each other by the configurations of the 3,4-diol centers. The small ax/ex coupling constant of H-4/H-3 (*J* = 3.0 Hz) in **2** suggested a *cis* relationship of C-3 and C-4 [9], which also suggested a half-chair conformation of the cyclohexenone ring. The 3*S*,4*R*-configuration was determined by comparison of its ECD spectrum (Figure 3) with that of (3*S*,4*R*)-4,8-dihydroxy-3-methoxy-3,4-dihydro-1(2*H*)-naphthalenone [9].

It should be noted that compounds **1** and **2** are diastereoisomers with the different ECD maxima at around 300–350 nm. The atomic spatial orientation and some little change of the half-chair conformation, especially the possible hydrogen bond between the *trans* or *cis* OH groups at C-3 and C-4 may effect the ECD spectra. In the literature [9,10], the absolute configurations at C-3 and C-4 of such naphthalenones were also determined by the Cotton effects at around 200–300 nm rather than above 300 nm.

Corynesidone E (**3**) was obtained as a white, amorphous powder. Its molecular formula was deduced as C_16_H_14_O_6_ by HRESIMS, thus revealing an increase in the molecular weight by 14 amu compared to the known compound corynesidone C, which was isolated from the endophytic fungus *C. cassiicola* L36, derived from the leaf tissues of the Chinese mangrove medicinal plant *Laguncularia racemosa*, collected from Hainan Island, China [11]. The ^1^H- and ^13^C-NMR spectra of both **3** and corynesidone C were very similar, except for the presence of signals for one methoxy group in the spectra of **3**. This was further confirmed by inspection of the HMBC spectrum revealing the correlations from 3-OCH_3_ to C-3 (Figure 2). Thus a methoxy group at C-3 was present in the structure of **3** instead of a hydroxy group at C-3 in corynesidone C. The structure of **3** was therefore determined as depict.

The structures of the known compounds, corynesidone A (**4**) [6], corynethers A (**5**) [6] and B (**6**) [7] were identified on the basis of their spectroscopic data and by comparison with those in the literature.

Compounds **1** and **2** were evaluated for their antifouling activities against the larval settlement of the barnacle *Balanus* *amphitrite*, but neither of the isolated compounds proved to be active. All of the isolated compounds were subjected to screening for their cytotoxic activities against the human promyelocytic leukemia HL-60, human leukemia K-562, human lung cancer A-549, and human cervical carcinoma HeLa cell lines. Only compound **4** exhibited moderate cytotoxic activity against HeLa cell line with an IC_50_ value of 15.2 μM. Compound **5** showed weak cytotoxic activity against HL-60 cells with IC_50_ values of 31.9 μM.

## 3. Experimental Section

### 3.1. General

Column chromatography (CC): silica gel (200–300 mesh; Qingdao Marine Chemical Group Co., Qingdao, China), octadecylsilyl silica gel (45–60 μm; Unicorn) and Sephadex LH-20 (Amersham Biosciences, Piscataway, NJ, USA). TLC: Precoated silica gel plates (G60, F-254; Yantai Zifu Chemical Group Co., Yantai, China). Prep. HPLC: Semi-preparative HPLC was performed on a Waters 1525 system coupled with a Waters 2996 photodiode array detector (Waters, Milford, MA, USA) using a C18 column (Kromasil, 5 μm, 10 × 250 mm). Optical rotations: JASCO P-1020 digital polarimeter (Jasco, Tokyo, Japan). UV spectra: Beckman DU 640 spectrophotometer (Beckman, Brea, CA, USA). ECD spectra: Jasco J-815-150S circular dichroism spectrometer (Jasco). IR spectra: Nicolet-Nexus-470 spectrometer (Nicolet, Glendale, WI, USA); KBr pellets; ν_max_ in cm^−^^1^. NMR spectra: Agilent DD2 500 MHz NMR spectrometer (500 MHz for ^1^H and 125 MHz for ^13^C), using TMS as an internal standard (Agilent, Santa Clara, CA, USA). HR-ESI-MS of compounds **1** and **2** were measured on a Bruker Q-TOF maXIS spectrometer (Bruker, Billerica, MA, USA). ESI-MS and HR-ESI-MS of compound **3** were obtained from a Micromass Q-TOF spectrometer (Waters) and Thermo Scientific LTQ Orbitrap XL spectrometer (Thermo, Waltham, MA, USA).

### 3.2. Fungal Material

The fungal strain *Corynespora cassiicola* XS-200900I7 was isolated from a piece of fresh tissue from the inner part of an unidentified sponge (XS-2009001), which was collected from the Xisha Islands coral reef in the South China Sea in December 2009. The Fungus was identified as *Corynespora cassiicola* according to its morphological characteristics and a molecular biological protocol by 16s rRNA amplification and sequencing of the ITS region. The strain was deposited in the Key Laboratory of Marine Drugs, the Ministry of Education of China, School of Medicine and Pharmacy, Ocean University of China, Qingdao, PR China, with the GenBank (NCBI) accession number KM597051.

### 3.3. Extraction, Isolation and Characterization

The fungal strain was cultivated in sixty Erlenmeyer flasks solid medium (each containing 80 g of rice, 120 mL of H_2_O, and 3.6 g natural sea salt from Yangkou saltern, China) at 28 °C for four weeks. The fermented solid medium was extracted three times with 400 mL of EtOAc for each Erlenmeyer flask. The combined EtOAc layers were evaporated to dryness under reduced pressure to give an EtOAc extract (18.6 g), which was subjected to vacuum liquid chromatography (VLC) on silica gel using step gradient elution with EtOAc–petroleum ether (0%–100%) and then with MeOH–EtOAc (0%–100%) to afford five fractions (Fr.1–Fr.5). Fr.2 was subjected to Sephadex LH-20 column chromatography (CH_2_Cl_2_/MeOH, *v*/*v*, 1:1) and further purified by using HPLC eluted with 60% MeOH–H_2_O to obtain **3** (5.0 mg), **4** (7.0 mg), and **5** (82.0 mg). Fr.3 was first subjected to repeated silica gel CC (CH_2_Cl_2_–MeOH, *v*/*v*, 100:1), and then separated by Sephadex LH-20 CC (CH_2_Cl_2_–MeOH, *v*/*v*, 1:1) to yield **6** (3.0 mg). Fr.4 was applied to repeated Sephadex LH-20 CC (CH_2_Cl_2_/MeOH, *v*/*v*, 1:1) and further purified on HPLC with 5% MeCN–H_2_O (with 0.1% trifluoroacetic acid) to give **1** (8.9 mg), and **2** (4.1 mg). The ^1^H-NMR, ^1^^3^C-NMR, COSY, HMQC, HMBC, MS spectra of **1**–**3** are available in Supplementary Materials.

*Corynenone A* (**1**)*:* brown powder; ${\left[\alpha \right]}_{D}^{20}$ = 19.4 (*c* 0.08, MeOH); UV (MeOH) λ_max_ (log ε) 221 (4.14), 290 (4.10); ECD (*c* 2.08 mM, MeOH) 215 (−5.51), 284 (+1.88); IR (KBr) ν_max_ 3648, 2319, 1646, 1542, 1340, 1021 cm^−1^; ^1^H- and ^13^C-NMR data, Table 1; HRESIMS *m*/*z* 239.0561 [M − H]^−^ (calcd for C_11_H_11_O_6_, 239.0561).

*Corynenone B* (**2**)*:* brown powder; ${\left[\alpha \right]}_{D}^{20}$ = 36.6 (*c* 0.40, MeOH); UV (MeOH) λ_max_ (log ε) 220 (4.31), 290 (4.24); ECD (*c* 2.08 mM, MeOH) 213 (+5.67), 284 (−2.75), 314 (+1.34); IR (KBr) ν_max_ 3607, 2364, 1697, 1536, 1447 cm^−1^; ^1^H- and ^13^C-NMR data, Table 1; HRESIMS *m/z* 239.0564 [M − H]^−^ (calcd for C_11_H_11_O_6_, 239.0561).

*Corynesidone E* (**3**)*:* white powder; UV (MeOH) λ_max_ (log ε) 230 (4.56), 274 (4.01); IR (KBr) ν_max_ 3431, 2950, 1634, 1414, 1266, 1117 cm^−1^; ^1^H-NMR (CDCl_3_, 500 MHz) δ 6.86 (1H, s, H-4), 6.52 (1H, s, H-7), 6.52 (1H, s, H-9), 3.95 (3H, s, H-13), 2.39 (3H, s, H-14), 2.30 (3H, s, H-12). ^13^C-NMR (CDCl_3_, 125 MHz) δ 163.5 (C-11), 156.4 (C-4a), 155.3 (C-8), 151.5 (C-3), 145.8 (C-9a), 143.8 (C-5a), 143.0 (C-2), 132.0 (C-6), 127.5 (C-1), 115.0 (C-11a), 114.3 (C-7), 105.7 (C-9), 101.7 (C-4), 56.7 (C-13), 16.1 (C-14), 13.3 (C-12). ESIMS: 303.2 [M + H]^+^, 325.2 [M + Na]^+^, 627.3 [2M + Na]^+^. HRESIMS *m*/*z* 303.0868 [M + H]^+^ (calcd for C_16_H_15_O_6_, 303.0863).

### 3.4. Biological Assays

The antifouling activity against the larval settlement of the barnacle was determined using cyprids of *Balanus amphitrite* Darwin based on the literature procedures [12]. SeaNine 211™ (Rohm & Haas) was used as a positive control. The cytotoxic activity was tested using four human tumour cell lines, including the human promyelocytic leukemia HL-60, human leukemia K-562, human lung cancer A-549, and cervical carcinoma HeLa. The cytotoxicity against HL-60 andK-562 was evaluated by MTT method [13], and the cytotoxicity against HeLa and A-549 was measured by SRB assay [14]. Adriamycin was used as a positive control.

## 4. Conclusions

In this study, three new compounds including two naphthalenones, corynenones A and B (**1** and **2**), and one depsidone, corynesidone E (**3**), together with three known compounds (**4**–**6**) were isolated from the sponge-derived fungus *Corynespora cassiicola* XS-20090I7. Their structures were determined by spectroscopic data and ECD spectra. Compounds **4** and **5** showed cytotoxicity against HL-60 and HeLa cell lines.

## Supplementary Materials

Supplementary materials can be accessed at: http://www.mdpi.com/1420-3049/21/2/160/s1.
