# Supplementary material for: Naphthalenones and Depsidones from a Sponge-Derived Strain of the Fungus Corynespora cassiicola
Source: Molecules. 2016 Jan 28;21(2):160. doi: 10.3390/molecules21020160 (PMC6273126; doi:10.3390/molecules21020160)
Supplement: Supplementary file 1 [file molecules-21-00160-s001.pdf]

Supplementary Materials: Naphthalenones and Depsidones from a Sponge-Derived Strain of the Fungus *Corynespora cassiicola*

Dong-Lin Zhao, Chang-Lun Shao, Chao-Yi Wang, Mei Wang, Lu-Jia Yang and Chang-Yun Wang

List of Supporting Information

Figure S1. ^{1}H-NMR (500 MHz, acetone-d_6) spectrum of compound **1**.
Figure S2. Partial ^{1}H-NMR (500 MHz, acetone-d_6) spectrum of compound **1**.
Figure S3. ^{13}C-NMR (125 MHz, acetone-d_6) spectrum of compound **1**.
Figure S4. HMQC (acetone-d_6) spectrum of compound **1**.
Figure S5. COSY (acetone-d_6) spectrum of compound **1**.
Figure S6. HMBC (acetone-d_6) spectrum of compound **1**.
Figure S7. HRESIMS spectrum of compound **1**.
Figure S8. ^{1}H-NMR (500 MHz, DMSO-d_6) spectrum of compound **2**.
Figure S9. Partial ^{1}H-NMR (500 MHz, DMSO-d_6) spectrum of compound **2**.
Figure S10. ^{13}C-NMR (125 MHz, DMSO-d_6) spectrum of compound **2**.
Figure S11. HMQC (DMSO-d_6) spectrum of compound **2**.
Figure S12. COSY (DMSO-d_6) spectrum of compound **2**.
Figure S13. HMBC (DMSO-d_6) spectrum of compound **2**.
Figure S14. HRESIMS spectrum of compound **2**.
Figure S15. ^{1}H-NMR (500 MHz, $CDCl_3$) spectrum of compound **3**.
Figure S16. ^{13}C-NMR (125 MHz, $CDCl_3$) spectrum of compound **3**.
Figure S17. HMQC ($CDCl_3$) spectrum of compound **3**.
Figure S18. HMBC ($CDCl_3$) spectrum of compound **3**.
Figure S19. ESIMS spectrum of compound **3**.
Figure S20. HRESIMS spectrum of compound **3**.

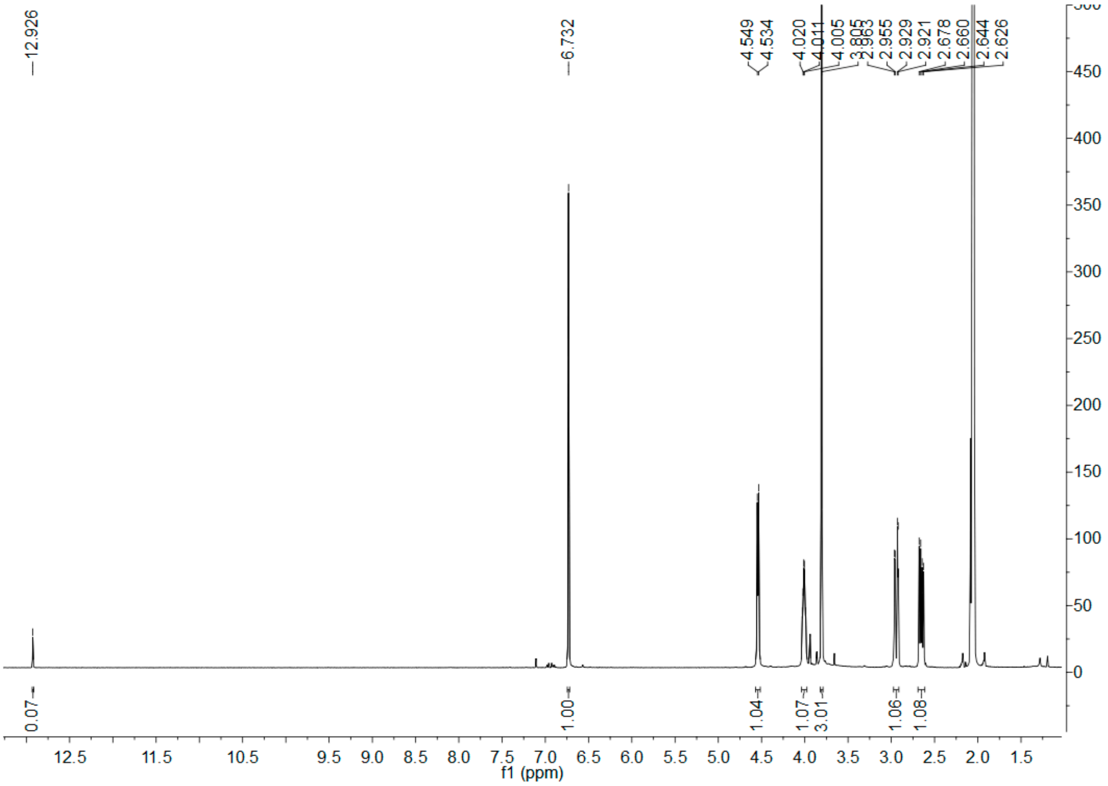

Figure S1. ^{1}H-NMR (500 MHz, acetone-d_6) spectrum of compound **1**.

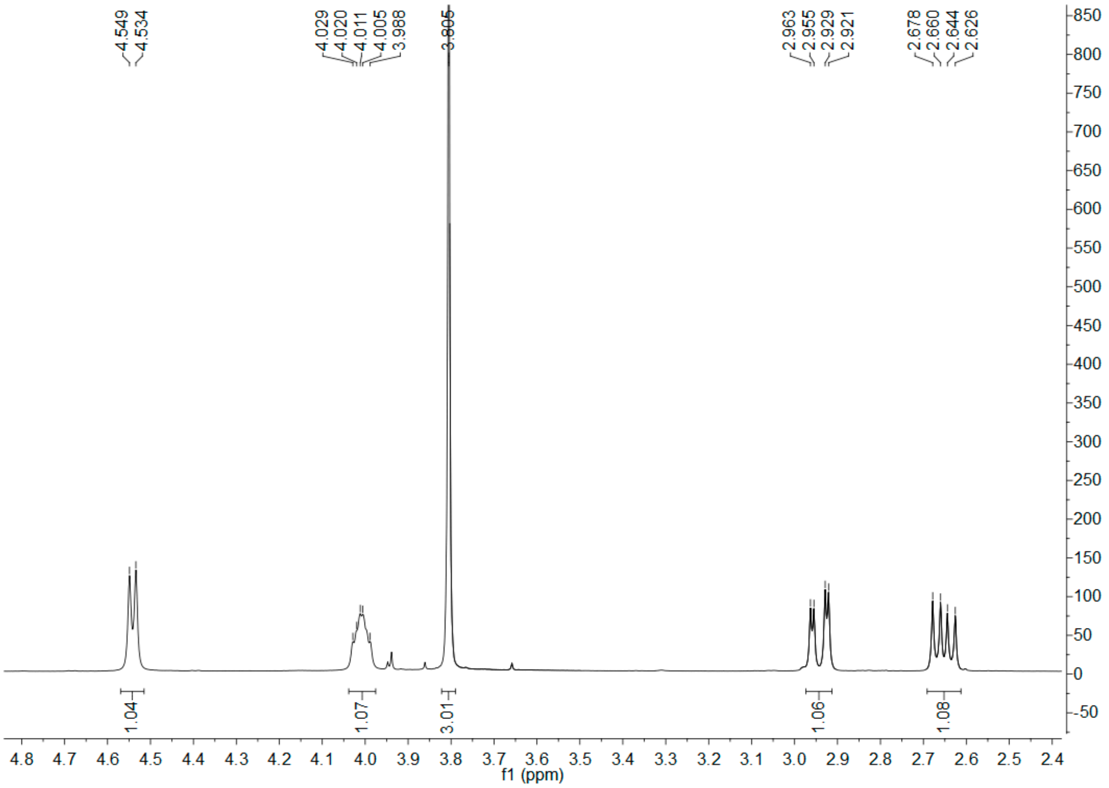

Figure S2. Partial ^{1}H-NMR (500 MHz, acetone-d_6) spectrum of compound **1**.

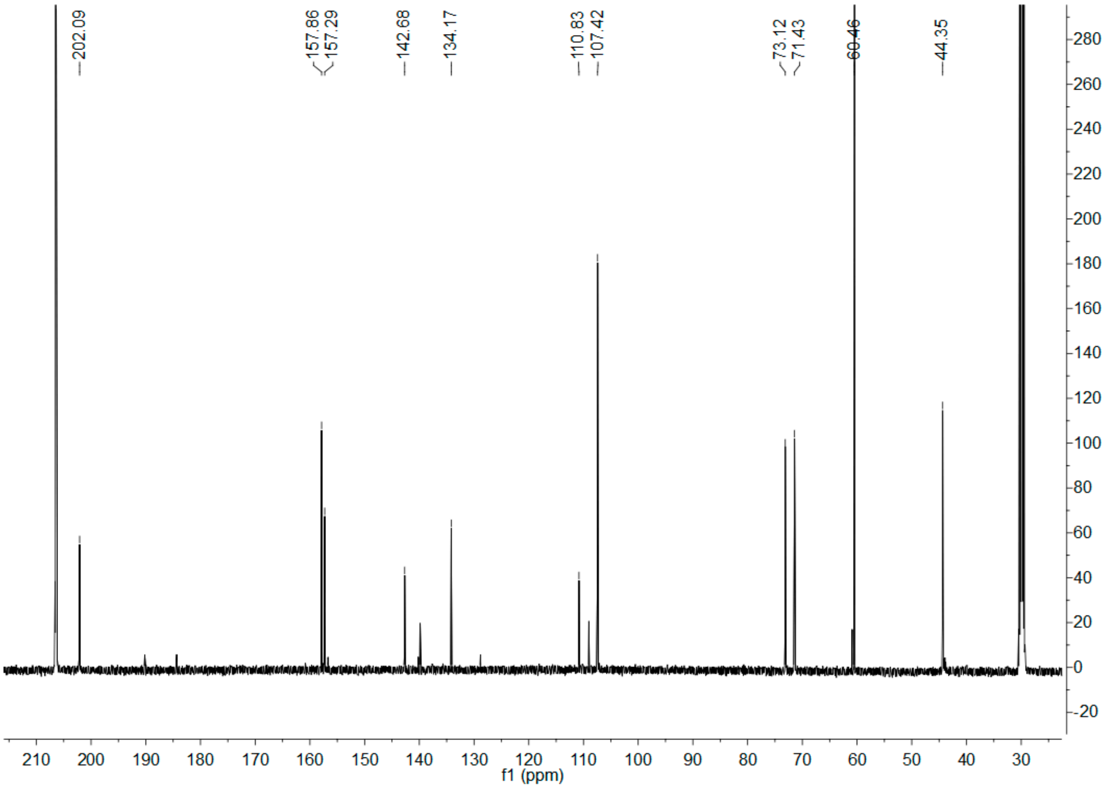

Figure S3. ^{13}C-NMR (125 MHz, acetone-d_6) spectrum of compound **1**.

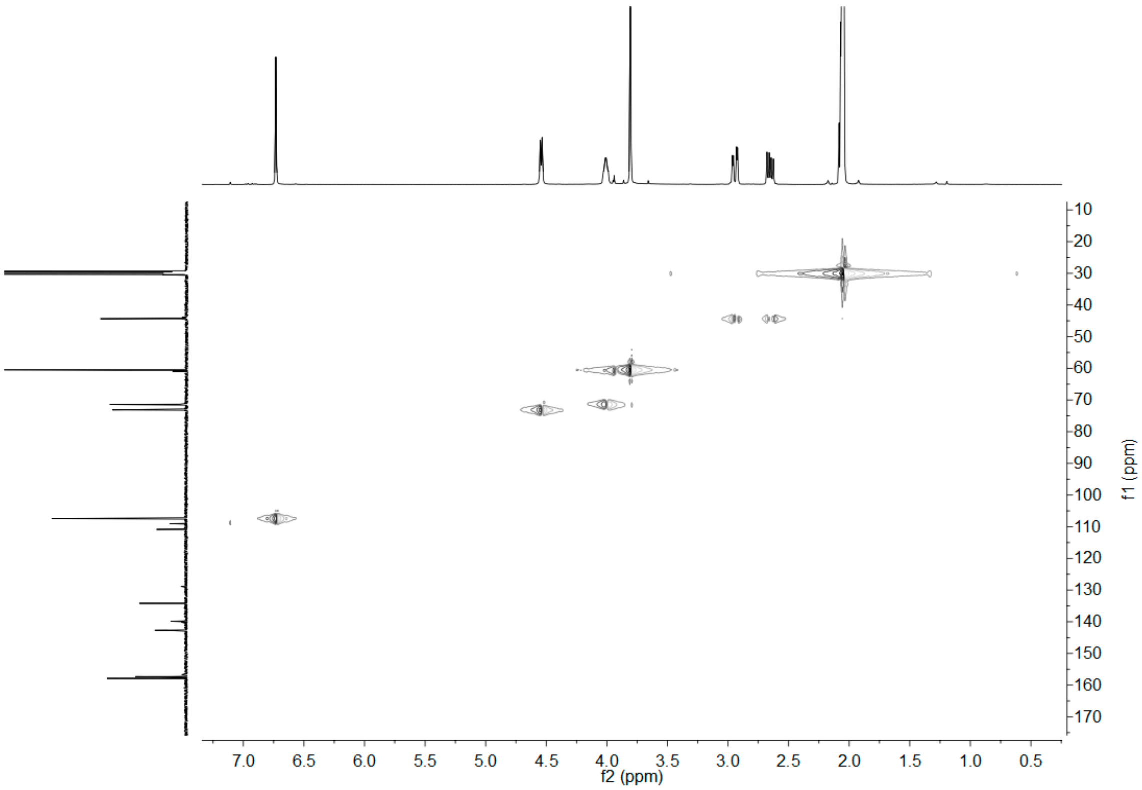

Figure S4. HMQC (acetone-*d*6) spectrum of compound **1**.

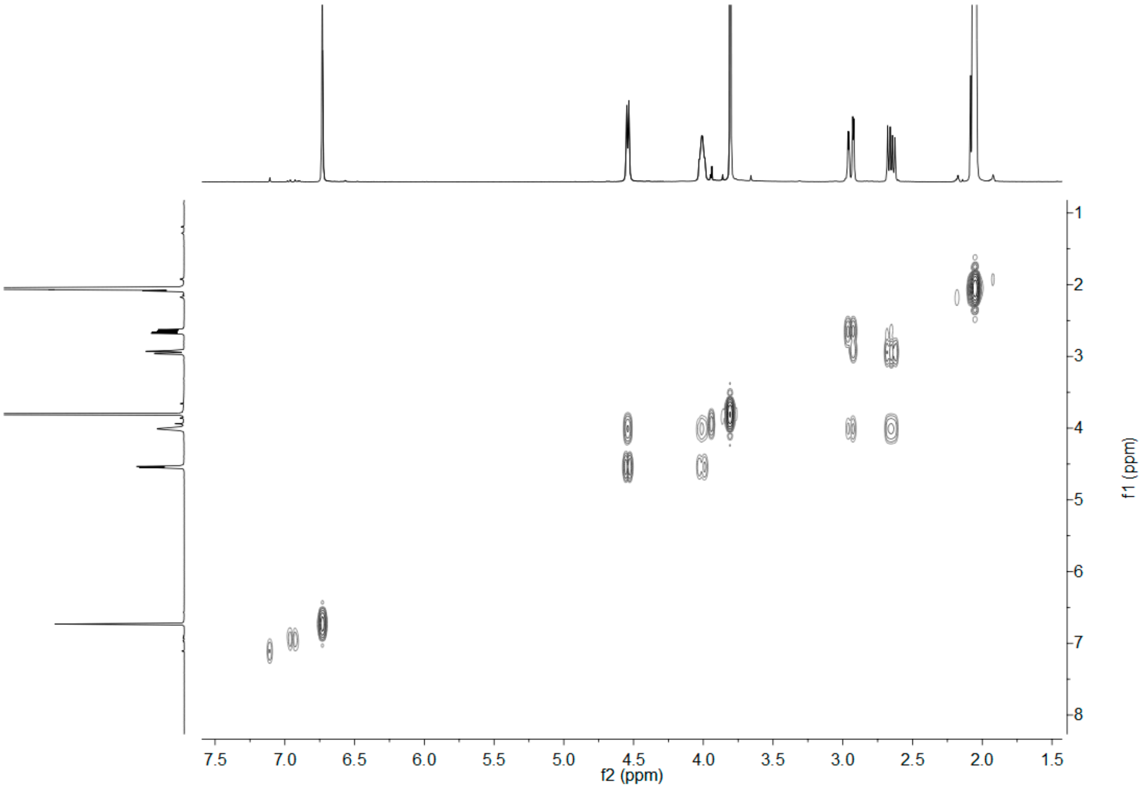

Figure S5. COSY (acetone-*d*6) spectrum of compound **1**.

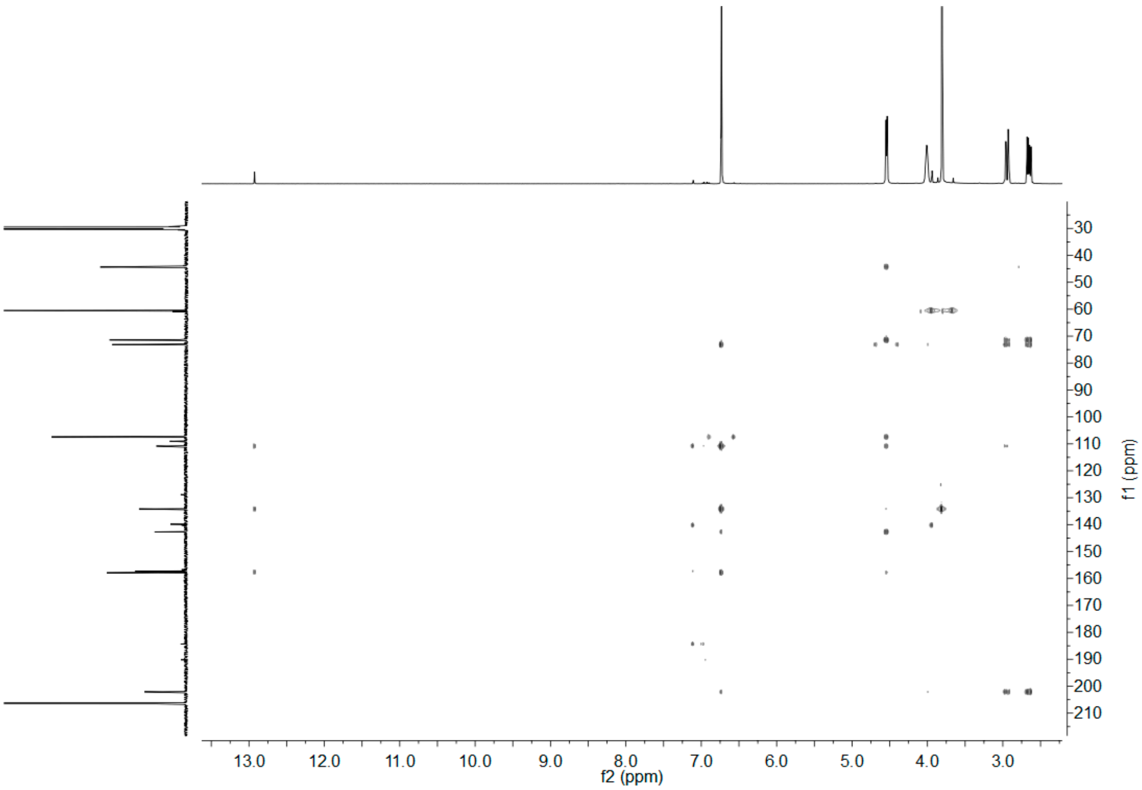

Figure S6. HMBC spectrum (acetone-*d*6) of compound **1**.

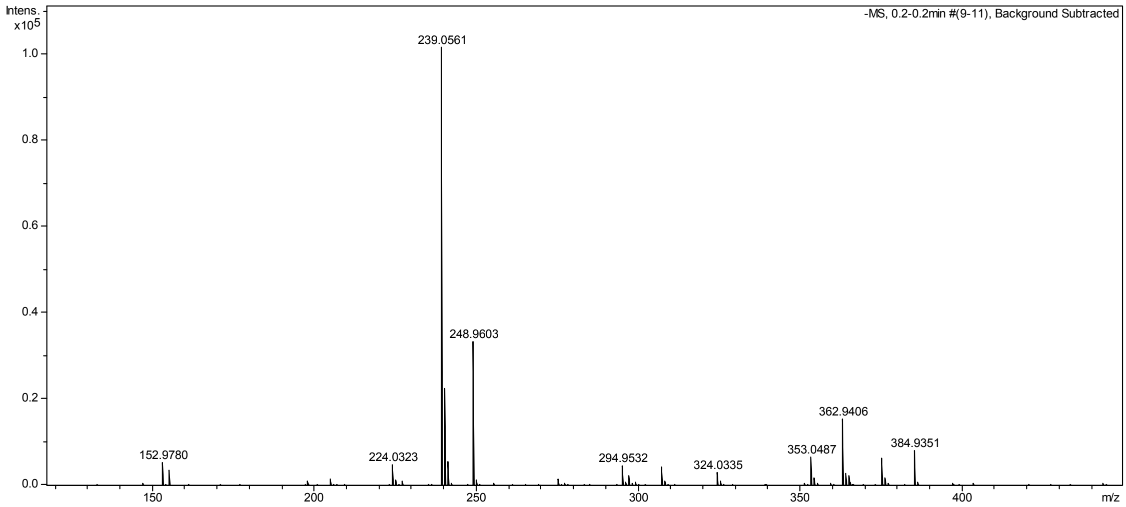

Figure S7. HRESIMS spectrum of compound **1**.

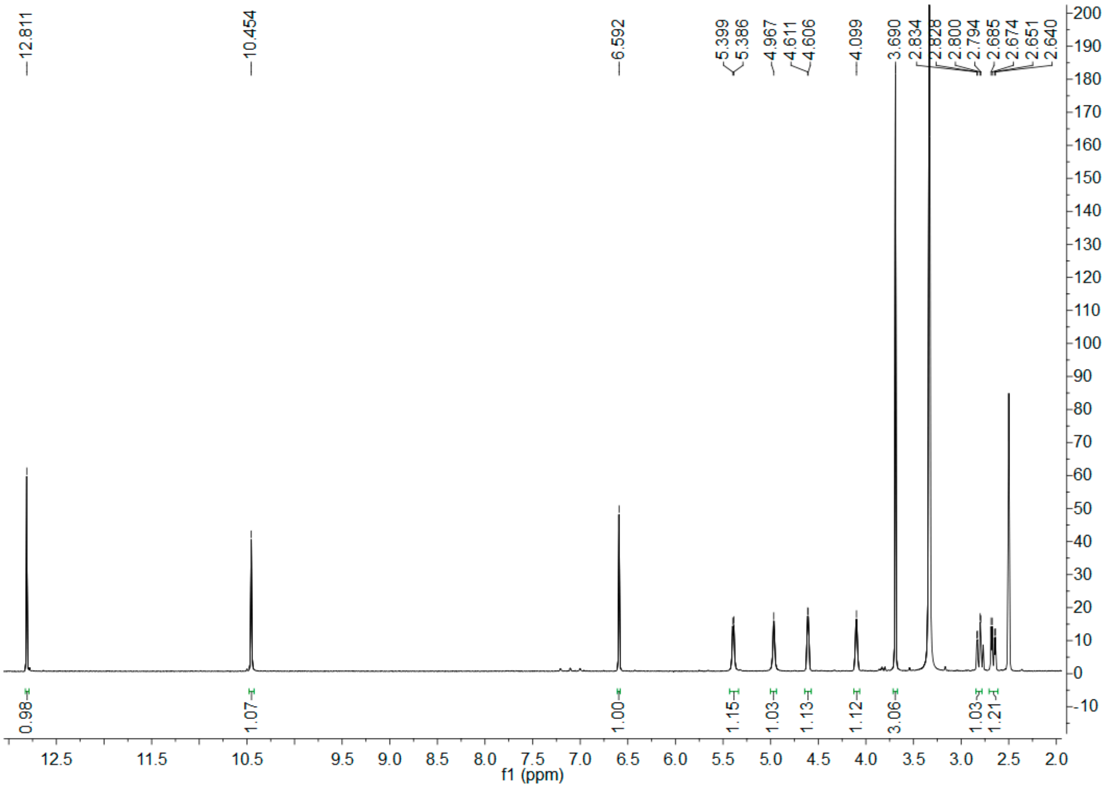

Figure S8. ^{1}H-NMR (500 MHz, DMSO-*d*$_6$) spectrum of compound **2**.

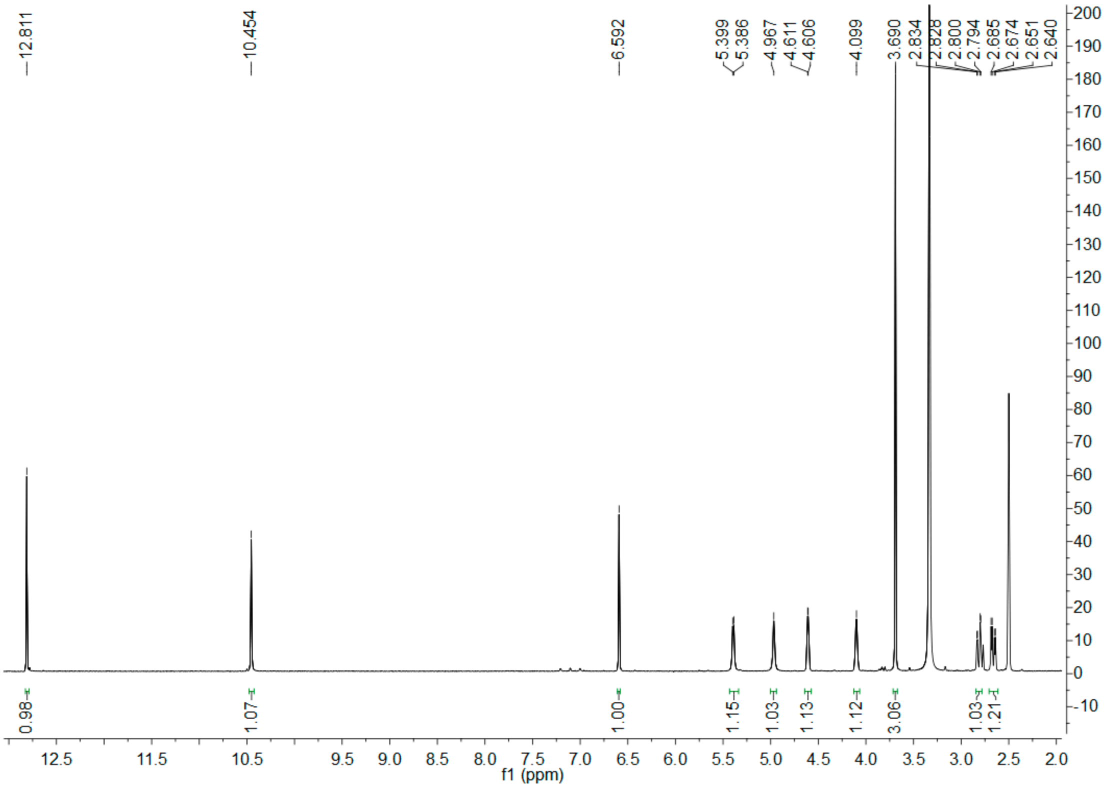

Figure S9. Partial ^{1}H-NMR (500 MHz, DMSO-*d*$_6$) spectrum of compound **2**.

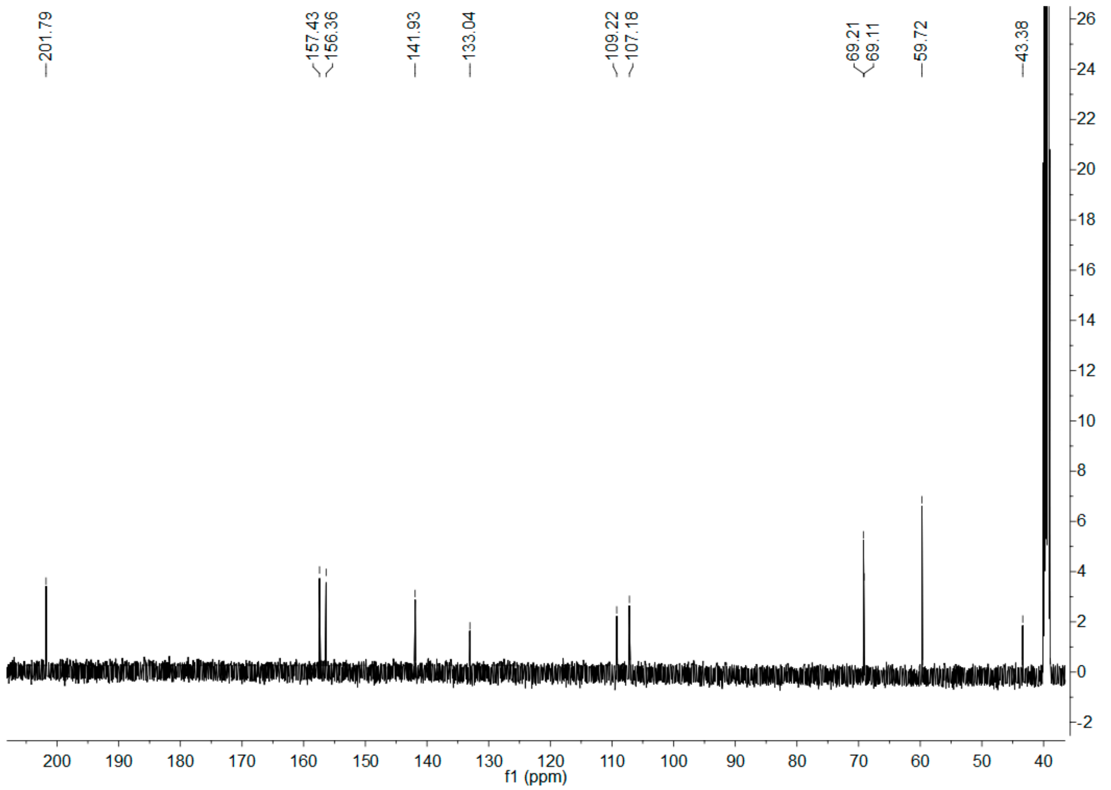

Figure S10. ^{13}C-NMR (125 MHz, DMSO-d_6) spectrum of compound **2**.

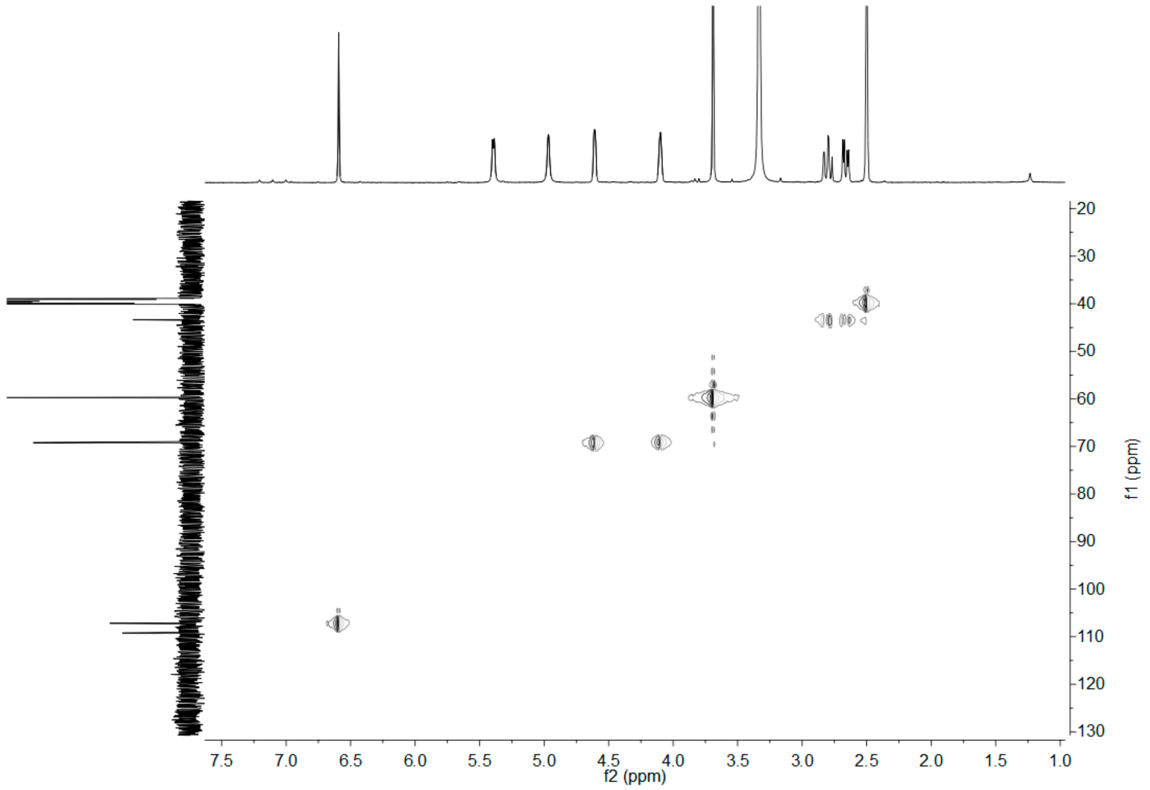

Figure S11. HMQC (DMSO-d6) spectrum of compound **2**.

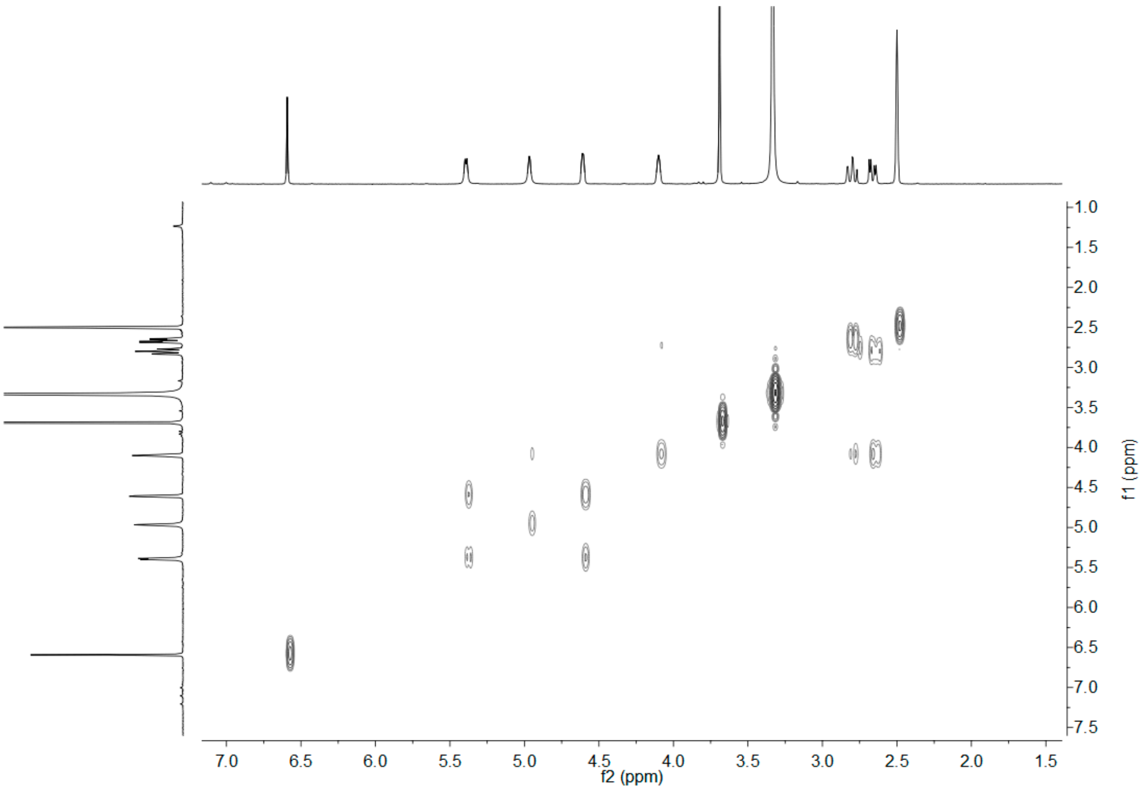

Figure S12. COSY (DMSO-d_6) spectrum of compound **2**.

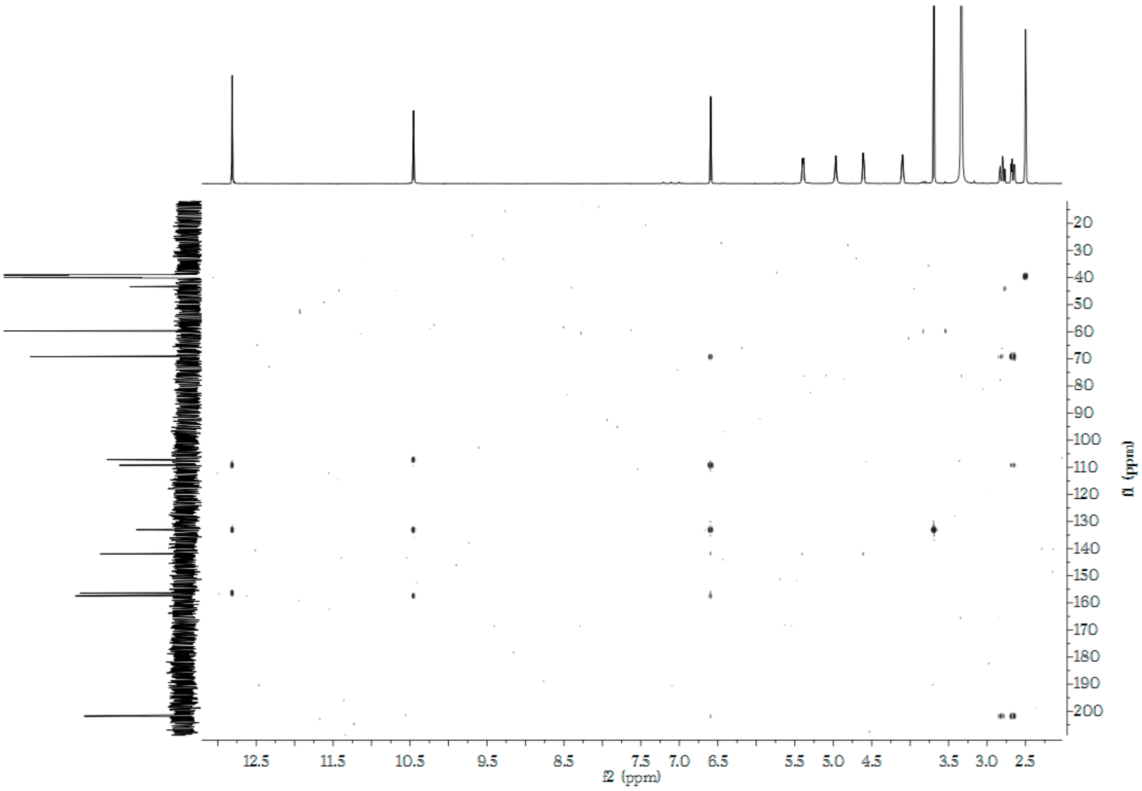

Figure S13. HMBC (DMSO-d_6) spectrum of compound **2**.

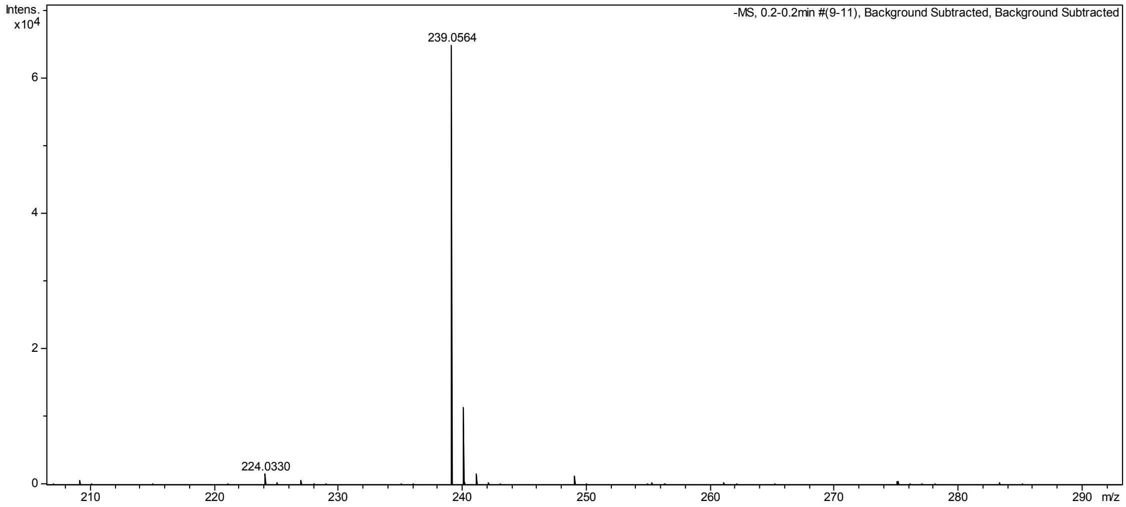

Figure S14. HRESIMS spectrum of compound **2**.

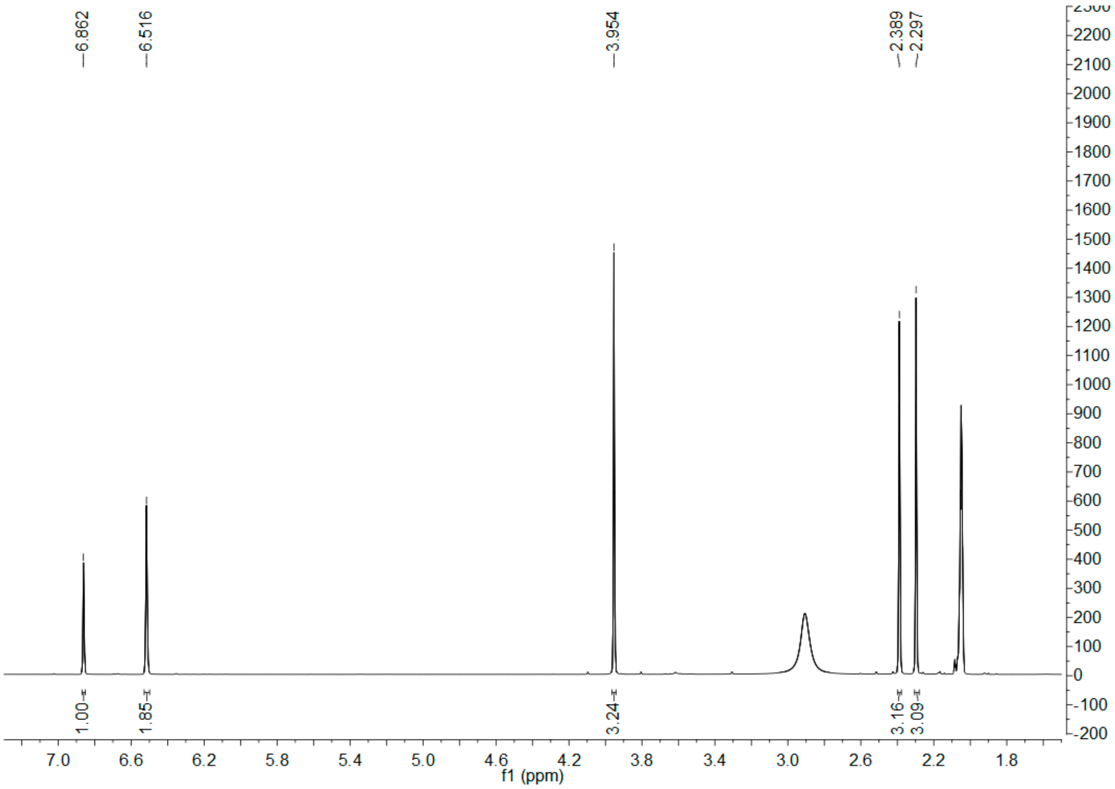

Figure S15. ¹H-NMR (500 MHz, $CDCl_3$) spectrum of compound **3**.

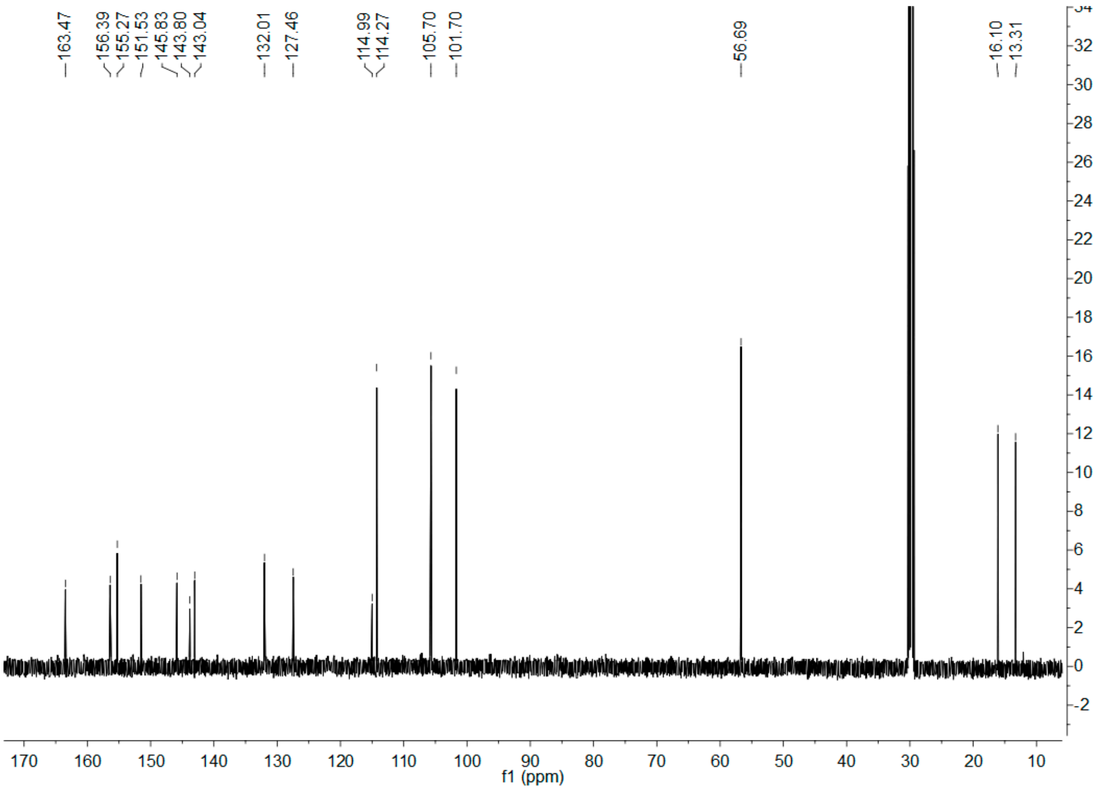

Figure S16. ^{13}C-NMR (125 MHz, $CDCl_3$) spectrum of compound **3**.

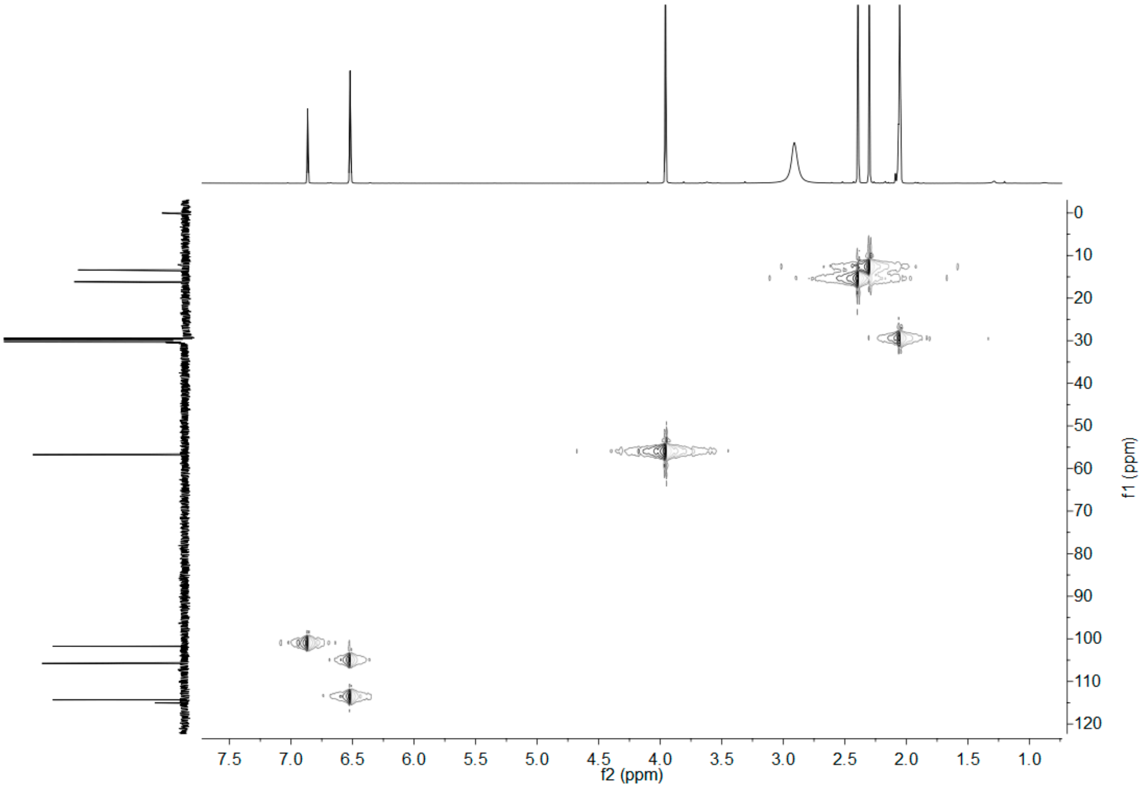

Figure S17. HMQC ($CDCl_3$) spectrum of compound **3**.

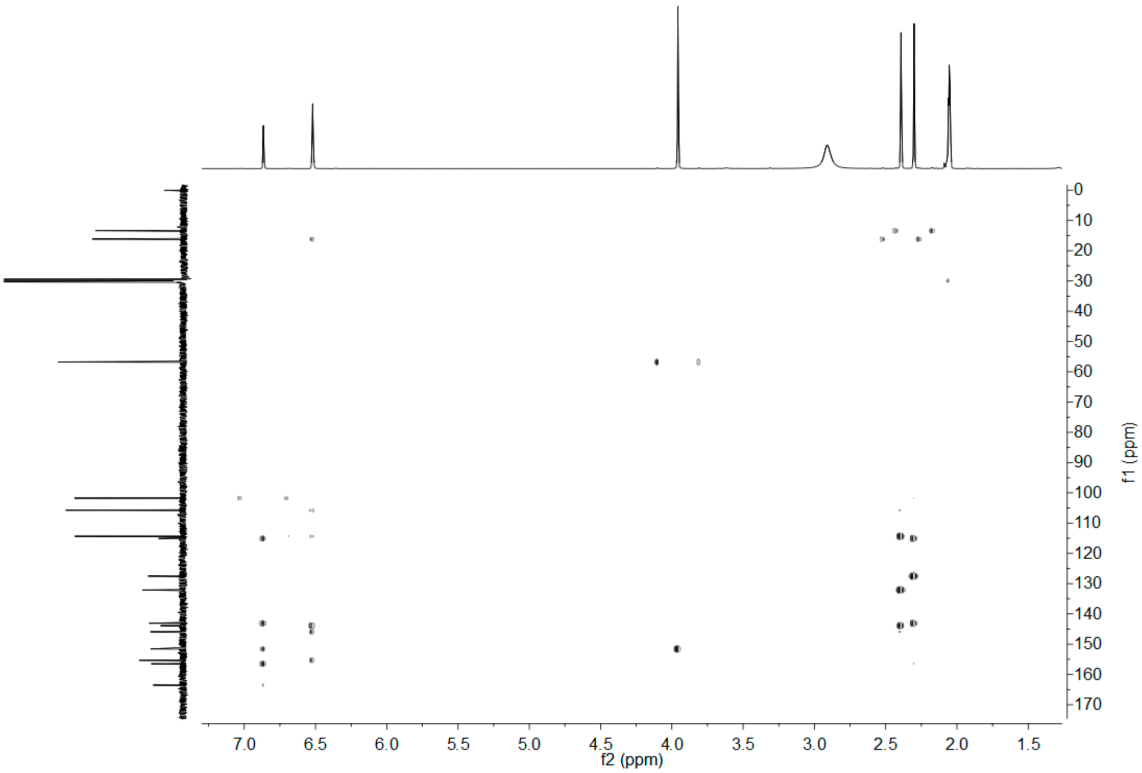

Figure S18. HMBC ($CDCl_3$) spectrum of compound **3**.

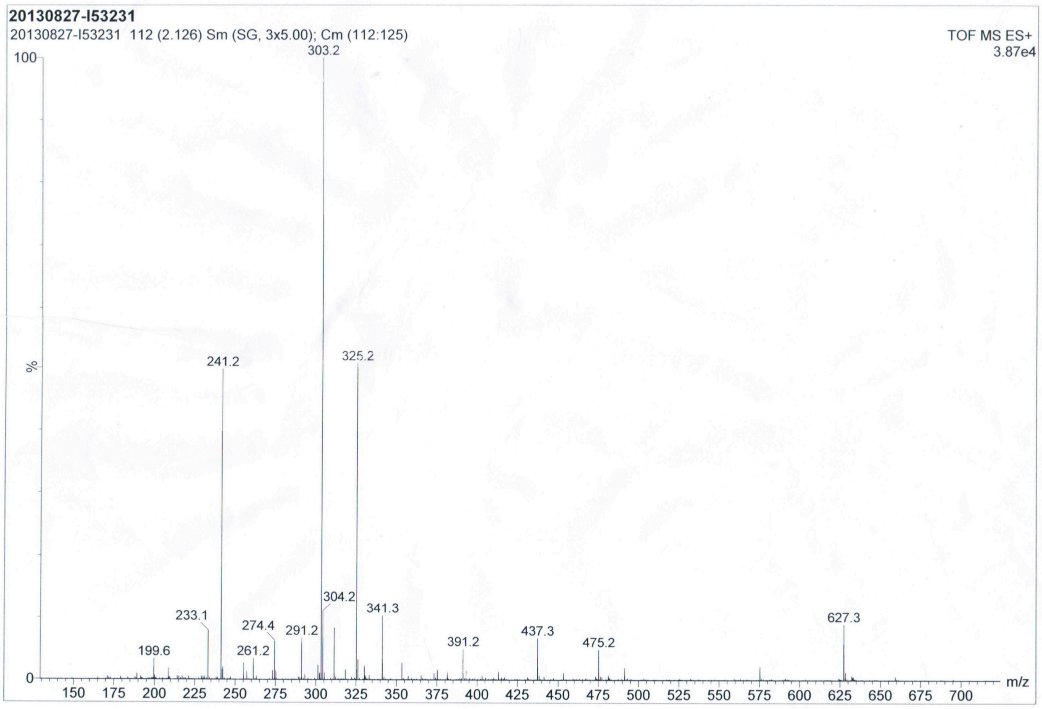

Figure S19. ESIMS spectrum of compound **3**.

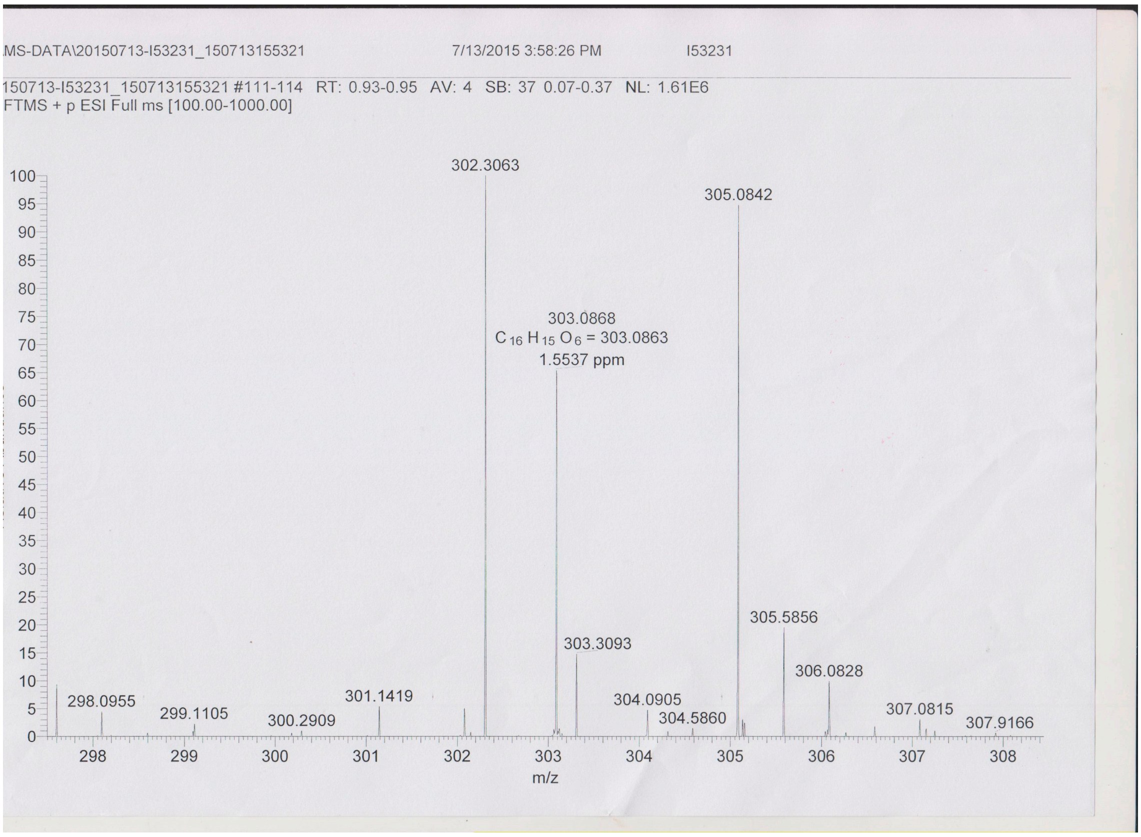

Figure S20. HRESIMS spectrum of compound **3**.
